# Raman scattering spectroscopic analysis on the structural changes in the micelles of a CTAB-Na*p*TS system

**DOI:** 10.1007/s44211-026-00950-w

**Published:** 2026-08-05

**Authors:** Ishige Yuki, Kenichi Oguchi, Hiroharu Yui

**Affiliations:** 1 Department of Chemistry, Faculty of Science, Tokyo University of Science, 1-3 Kagurazaka, Shinjuku, Tokyo, 162-8601 Japan; 2 Water Frontier Research Center, Research Institute for Science & Technology, Tokyo University of Science, 1-3 Kagurazaka, Shinjuku, Tokyo, 162 8601 Japan

**Keywords:** Raman scattering, Micelle, CTAB, Salt

## Abstract

Condensed micelle solutions are ubiquitously seen and utilized in foods, medicine, oil recovery, and so on. It is well-known that micelles formed from ionic surfactants drastically change their aggregate morphologies from spherical to short, rigid rod-like, and occasionally to long, winding worm-like structures as the concentration of the co-existing salts is varied. In general, these large-scale morphological changes induced by additive salt have been directly assessed by X-ray or neutron scattering spectroscopies. However, industrial processes are often carried out under open-system and atmospheric conditions, thus they show transient and dynamic changes of their structures and resultant viscoelastic properties according to the environmental changes. Here we examined whether Raman scattering spectroscopy is applicable for detecting such large-scale morphological changes in micelles by monitoring the corresponding changes occurring in the local packing environment of neighboring surfactant alkyl chains. As a representative micelle-salt system, cetyltrimethylammonium bromide (CTAB) and sodium *p*-toluene sulfonate (Na*p*TS) were studied by varying their concentration ratio. As a result, it was proposed that the CH_2_ twisting and scissoring modes were useful indices to discriminate the morphological changes in micelles. These results provide a useful index for monitoring the transient structural changes of micelle solutions and elucidating the mechanism of the resultant changes in viscoelastic properties in situ and on-site monitoring.

**Graphical abstract:**

**Figure:**
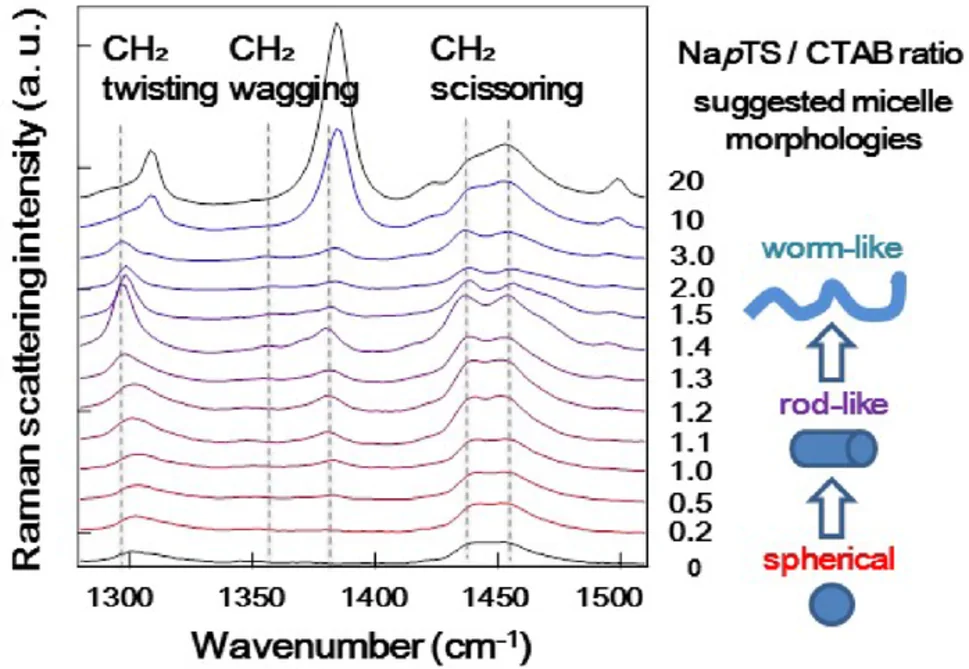

## Introduction

Aqueous solutions composed of condensed micelle-salt systems are ubiquitous in foods, pharmaceuticals, detergents, oil recovery and exhibit various viscoelastic properties such as shear thinning and thickening [1–5]. Controlling the viscoelastic properties of micellar solutions, including shear thickening and thinning, is crucial for achieving efficient processing and desirable final product textures in a wide range of industrial applications. The morphology of the micelles themselves, as well as their spatial packing and entanglement, plays a crucial role in determining these viscoelastic properties [6–11]. Micelle morphology generally varies such as spherical, rod-like, worm-like, and lamella structures. Because the viscoelastic properties of the micellar solution depend on these morphological changes, methods for identifying the various micellar structures are essential for understanding and controlling their rheological behaviors. In general, small angle X-ray scattering (SAXS) and small angle neutron scattering (SANS) have been widely used to discriminate among different micellar structures in solution [12–20]. These scattering techniques directly probe large-scale aggregate structures and provide powerful tools for distinguishing among various micellar morphologies. However, these measurements are generally performed in closed systems and, in some cases, under vacuum conditions to ensure efficient transmission of X-Ray and neutron beams by using large-scale instrumentation.

In contrast, in industrial applications of condensed micelle-salt aqueous solutions, these systems are generally used under open-system and ambient conditions in the presence of humidity and additives such as salts and oils. Under these conditions, the micelle structures are expected to undergo dynamic changes due to the evaporation of water molecules and/or interactions with salt and oil molecules. Such transient changes of the micellar morphologies often results in changes of viscoelastic properties of the solution. In particular, under flow conditions, these changes in micellar morphology can induce shear thickening and thinning [21]. These transient changes in viscoelastic properties are important for controlling the efficiency of the industrial processes and the perfomance of final products, such as enhanced oil recovery, inkjet printing, and food texture. However, it is generally difficult to monitor such transient changes in micelle morphology in real-time under open-system and ambient conditions.

Here we examined the applicability of the Raman scattering spectroscopy for monitoring such morphological transitions of micelle structures by probing changes in the local environments of neighboring alkyl chains. As a model detergent-salt sample, cetyltrimethylammonium bromide (CTAB) and sodium *p*-toluene sulfonate (Na*p*TS) were selected because the structural transitions of the CTAB-Na*p*TS micelles have been extensively investigated by SANS. Previous studies have shown that the structural changes from spherical to short rod-like and worm-like micelles as the concentration ratio of the detergent (*C*_D_) to the added salt (*C*_S_) is varied [21]. However, the accompanying changes in the local packing environments and structural distortion of alkyl chains during morphological transitions remain unclear particularly as the Na*p*TS/CTAB molecular ratio molecular ratios (*C*_S_/*C*_D_) changes. If the local spatial packing of neighboring alkyl chains varies in concert with the large-scale morphological changes of the micelles, Raman spectroscopy should enable the detection and real-time monitoring of such structural transitions, even under open-system conditions and in the presence of various additives.

Raman spectroscopic studies of CTAB have previously been reported, and the assignments of its various vibrational modes have been established [22, 23]. In general, the local changes in the packing environments of alkyl chains have evaluated by changes in the intensity ratio of the trans and gauche conformations observed in the C-C stretching and/or CH_2_ stretching modes. This approach is effective for pure surfactant system. However, analyses based on the intensity ratios of Raman bands often suffer from the interference by Raman signals arising from additives, such as salts and oils, making quantitative evaluation of the target signals difficult. Therefore, in the present study, we focused on the applicability of the wavenumber shifts and bandwidths of the Raman bands as indicators of changes in the local packing of surfactant alkyl chains in different micellar morphologies. Unlike intensity-based analyses, the wavenumber and bandwidth of Raman band are essentially independent of the relative concentrations of the surfactant and additives in the system. The present results show that the wavenumber and bandwidth of CH_2_ twisting and scissoring modes are highly sensitive to changes in the local packing environments of alkyl chains. These spectroscopic features therefore provide useful indicators for discriminating morphological changes in micelles induced by variations in the detergent-to-additive ratio.

## Experimental

Cetyltrimethylammonium bromide (CTAB, ca. 98%, MP Biomedicals) and *p*-toluenesulfonic acid sodium salt (Na*p*TS, > 85.0%, FUJIFILM Wako Pure Chemical Corporation) were utilized as received. The critical micelle concentration (cmc) of CTAB in water is approximately 1 mM at room temperature [24]. The concentration of CTAB in the aqueous solution examined in the present study was fixed at 50 mM, ensuring the formation of a sufficient number of micelles. Ultrapure water was prepared using a Milli-Q system (> 18.0 MΩ·cm; Simplicity UV, Millipore Co. Ltd.) to minimize contamination by dissolved salts. Because the CTAB concentration was more than 50 times of the cmc, the observed changes in the Raman spectra were expected to mainly reflect changes in the spatial packing of alkyl chains within micelles exhibiting different morphologies such as spherical, short rod-like, and worm-like structures. The concertation of the additive salt (Na*p*TS) was varied over a wide range to induce structural transitions of the CTAB micelles up to a *C*s/*C*d = 20. The CTAB-Na*p*TS aqueous solutions were prepared by mixing the components at 40 °C and allowing the mixtures to equilibrate overnight.

Raman scattering spectra of the CTAB-Na*p*TS aqueous solutions were recorded using a conventional Raman microscope system (Raman-11i, Nanophoton Co. Ltd.) at room temperature (20 °C). The excitation wavelength was 532 nm, and the laser power was 50 mW. The acquisition time for each Raman spectrum was 200 s, and no spectral accumulation was performed. A Nikon Plan Fluor objective lens (20×, NA = 0.50) was used for all measurements. The slit width of the spectrometer was set to 50 μm. The wavenumber axis of the spectra was calibrated using a certified polystyrene Raman standard (NMIJ RM 8158-a, AIST) with four reference bands at 620.7 cm^− 1^, 1001.2 cm^− 1^, 1031.5 cm^− 1^, and 1602.1 cm^− 1^. The spectral baseline was corrected over the wavenumber range of 1270–1515 cm^− 1^ by subtracting a linear function. A diffraction grating with 600 lines mm^− 1^ was used, providing a spectral dispersion was 1.9 cm^− 1^ per CCD pixels.

To minimize water evaporation during the measurements, the sample solutions were sandwiched between two cover glasses (thickness: 0.12–0.17 mm; Matsunami Glass Co. Ltd.). Each sample was measured four times under identical conditions and the spectra obtained were analyzed by Igor pro 8 software (WaveMetrics).

## Results and discussion

**Fig. 1:**
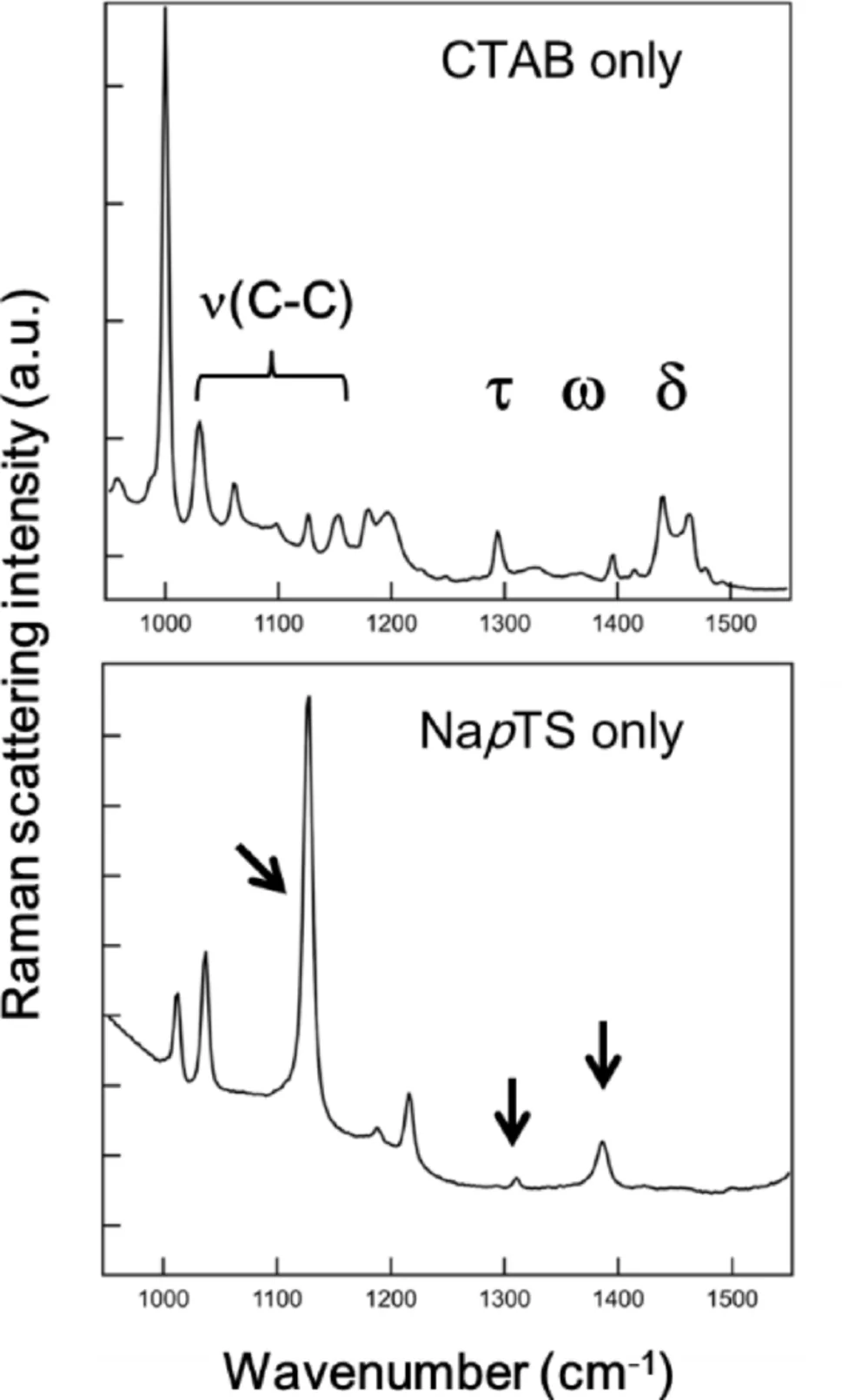
Comparison of the Raman spectra of pure CTAB (upper panel) and NapTS (lower panel) in the fingerprint region. The characteristic vibrational modes of the alkyl chains are indicated: C-C stretching in the liquid phase (ν, ~ 1100 cm^− 1^), twisting (τ,~1300 cm^− 1^), wagging (ω,~1380 and ~ 1360 cm^− 1^), and scissoring (δ, ~ 1450 cm^− 1^). The arrows in the lower panel indicate Na*p*TS peaks that should be taken in account when analyzing the spectra of the CTAB alkyl chains, because these peaks overlap with the CTAB bands as the Na*p*TS concentration increases

First, Raman scattering spectra of the CTAB and Na*p*TS were recorded separately to evaluate the possible spectral interference between the Raman bands of the two compounds (Fig. 1). For CTAB, the C-C stretching (ν, at around 1100 cm^− 1^), twisting (τ, 1300 cm^− 1^), wagging (ω, 1380 and 1360 cm^− 1^), and scissoring (δ, 1450 cm^− 1^) modes of the alkyl chains were clearly observed (upper panel in Fig. 1). These bands are in good accordance with those previously reported for CTAB micellar solutions [22, 23]. In contrast, Na*p*TS exhibited strong Raman bands, including the SO_3_^−^ stretching modes. The arrows in the lower panel of Fig. 1 indicate the bands that require particular attention during the spectral analysis of the CTAB alkyl-chain vibrations because they partially overlap with the CTAB Raman bands as the molecular ratio of Na*p*TS/CTAB increases. The strongest band, observed at 1125 cm^− 1^, is assigned to the S-O stretching mode [25, 26]. Because Na*p*TS contains a methyl (−CH_3_) group in its molecular structure, its Raman bands may also interfere with the CH_2_ vibrational region of the long alkyl chains of CTAB, particularly at high *C*s / *C*d ratios (> 10). In the present study, the possible spectral interference arising from the Na*p*TS Raman bands indicated by the arrows in the lower panel of the Fig. 1 was carefully considered.

**Fig. 2:**
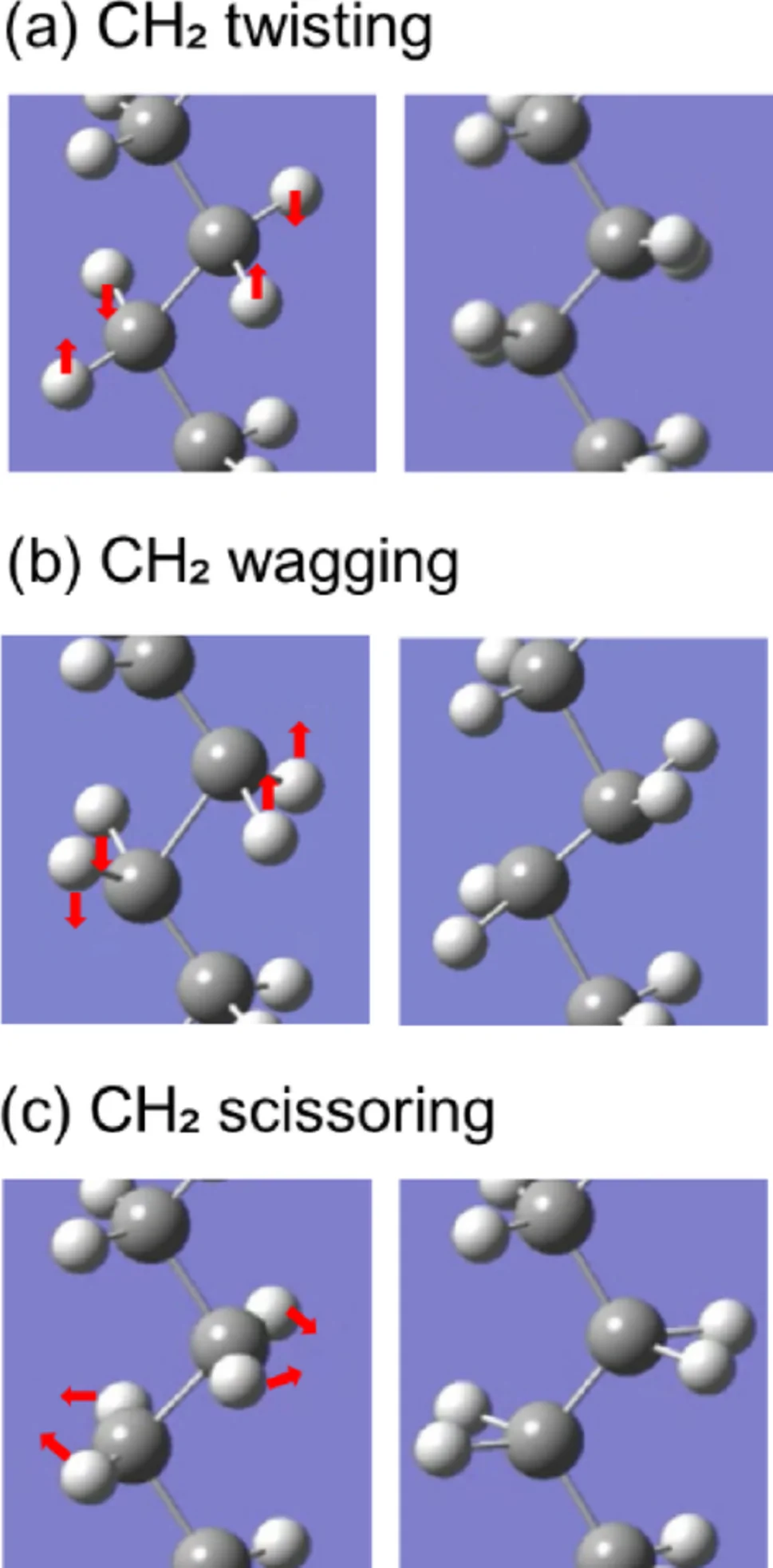
Schematic illustrations of the CH_2_ vibrational modes: **a** twisting (τ, ~ 1300 cm^− 1^) **b** wagging (ω, ~ 1380 and ~ 1360 cm^− 1^), and **c** Scissoring (δ, ~ 1450 cm^− 1^). The twisting and wagging modes involve vibrations parallel to the axis of the C-C skeletal structure, whereas the scissoring mode involves vibrations in the radial (perpendicular) direction relative to the axis of the C-C skeletal axis

In general, changes in the local packing environments of alkyl chains have been investigated using three characteristic Raman spectral regions: (i) the low-wavenumber longitudinal acoustic modes (100–300 cm^− 1^); (ii) the skeletal C–C stretching and methylene rocking modes (1000–1200 cm^−1^); and (iii) the C-H stretching modes (2800–3000 cm^− 1^) [27]. In particular, for surfactant alkyl chains in micellar aggregates, steric packing constraints are expected in the core of spherical micelles and around the central axis of rod-like and worm-like micelles, where substantial bending of the alkyl chain is likely to occur. Such structural constraints are expected to alter the trans/gauche conformational equilibrium. Accordingly, the skeletal C–C stretching vibrations ν (C–C) in the 1000–1200 cm^− 1^ region have long been used to evaluate conformational changes through variation in the intensity ratio of the trans and gauche conformers [28]. The bending of alkyl chains accompanying the morphological transition of micelles is expected to influence not only the trans / gauche intensity ratio but also affect the wavenumber and bandwidth of the CH_2_ twisting, wagging, and scissoring modes through the distortion of the C–C backbone (Fig. 2). Indeed, these vibrational modes have previously been employed to analyze mixtures of ceramides and saturated fatty acids [29]. A major advantage of using the CH_2_ twisting, wagging, and scissoring modes is that the analyses is based on the wavenumber shifts and bandwidth changes rather than on intensity ratio. Consequently, this approach is expected to be more robust against variations in the concentrations of additives and changes in solvent concentration, conditions that are commonly encountered in industrial processes such as food and pharmaceutical manufacturing, inkjet printing, and enhanced oil recovery.

**Fig. 3:**
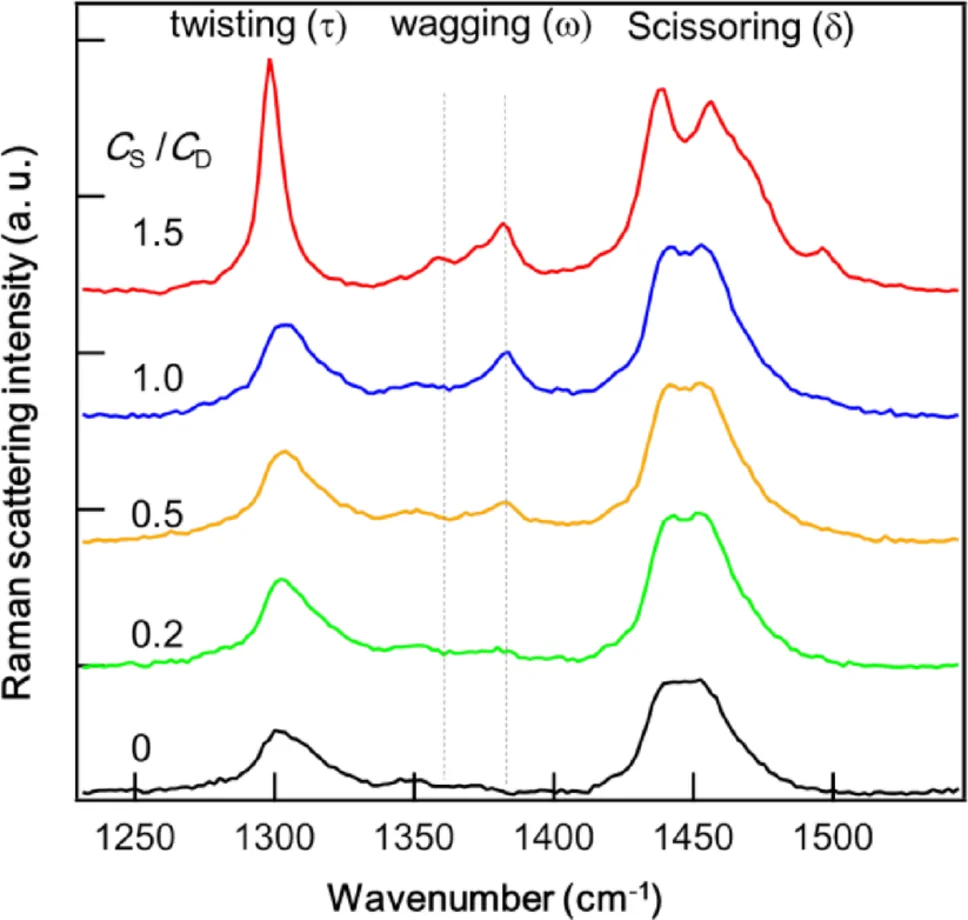
Comparison of the Raman spectra of the CTAB-Na*p*TS system at *C*_S_/*C*_D_ ratios of 0, 0.2, 0.5, 1.0, and 1.5. Progressive sharpening of the CH_2_ twisting band and more distinct splitting of the CH_2_ scissoring band are observed with increasing *C*_S_/*C*_D_, particularly at *C*_S_/*C*_D_ = 1.5. The two dotted lines indicate the expected wavenumbers of the CH_2_ wagging modes at 1360 cm^− 1^ and 1380 cm^− 1^

We applied the Raman scattering spectroscopy focusing on the twisting, wagging, and scissoring modes to discriminate the expected morphologies of CTAB-Na*p*TS (surfactant – salt) system and to evaluate the effectiveness of these vibrational modes as structural probes. First, we measured Raman spectra of CTAB-Na*p*TS system by varying the concentration ratios of the salt (*C*s, Na*p*TS) to surfactant (*C*_D_, CTAB), with *C*_S_/*C*_D_ values of 0, 0.2, 0.5, 1.0, and 1.5 (Fig. 3). A single band was observed at low *C*_S_/*C*_D_, whereas emergence of splitting of the CH_2_ scissoring (δ) mode was observed and clear splitting was finally evident at *C*_S_/*C*_D_ = 1.5. Previous studies using SANS and static light scattering (SLS) have reported that the CTAB-Na*p*TS system forms spherical micelles at *C*_S_/*C*_D_ = 0–0.5, undergoes a transition from the spherical to rod-like structures at *C*_S_/*C*_D_ = 0.5–0.7, and predominantly forms rigid rod-like micelles above *C*_S_/*C*_D_ > 0.7 [21]. Thus, the gradual changes observed in the Raman spectra of the CTAB-Na*p*TS system can be attributed to the structural transition from spherical to rigid rod-like micelles, as evidenced by the clear peaks splitting of the CH_2_ scissoring band at around 1450 cm^− 1^. In spherical micelles, the alkyl chains of CTAB are loosely packed in the hydrophobic core driven by hydrophobic interactions in aqueous solution. During the transition from the spherical to rigid rod-like structures with increasing *C*_S_/*C*_D_, the packing of CTAB alkyl chains becomes progressively more ordered along the long axis of cylindrical region of rod-like micelles. Although both ends of rod-like micelles retain hemispherical caps, the increasing cylindrical region, where the alkyl-chain packing becomes more uniform, is expected to result in sharper Raman features compared with spherical micelles. The observed clear splitting of the CH_2_ scissoring band with increasing *C*_S_/*C*_D_ strongly supports the spherical-to-rod-like structural transition proposed by Takeda et al. [21].

To further examine this hypothesis, the changes in CH_2_ wagging (ω, 1380 cm^− 1^ and 1360 cm^− 1^) and twisting (τ, at around 1300 cm-1) modes of CTAB alkyl chains were also investigated. However, we were unable to clearly resolve the changes in either of two wagging bands. The band at 1380 cm^− 1^ overlaps with Raman signals from Na*p*TS, while the band at 1360 cm^− 1^ is intrinsically weak in intensity. In contrast, a clear red shift and band narrowing of the CH_2_ twisting mode (τ, around 1300 cm^− 1^) were observed with increasing *C*_S_/*C*_D_ up to 1.5. This blue-shift (indicating strengthened interaction between the neighboring alkyl chains, namely chain packing tightening) and sharpening (reflecting increased structural order) strongly support a transition from spherical packing to cylindrical micellar structures. These results demonstrate that the CH_2_ twisting mode in Raman spectra can be effectively used to discriminate the morphological transition between from spherical to ridged rod-like cylindrical micelles.

**Fig. 4:**
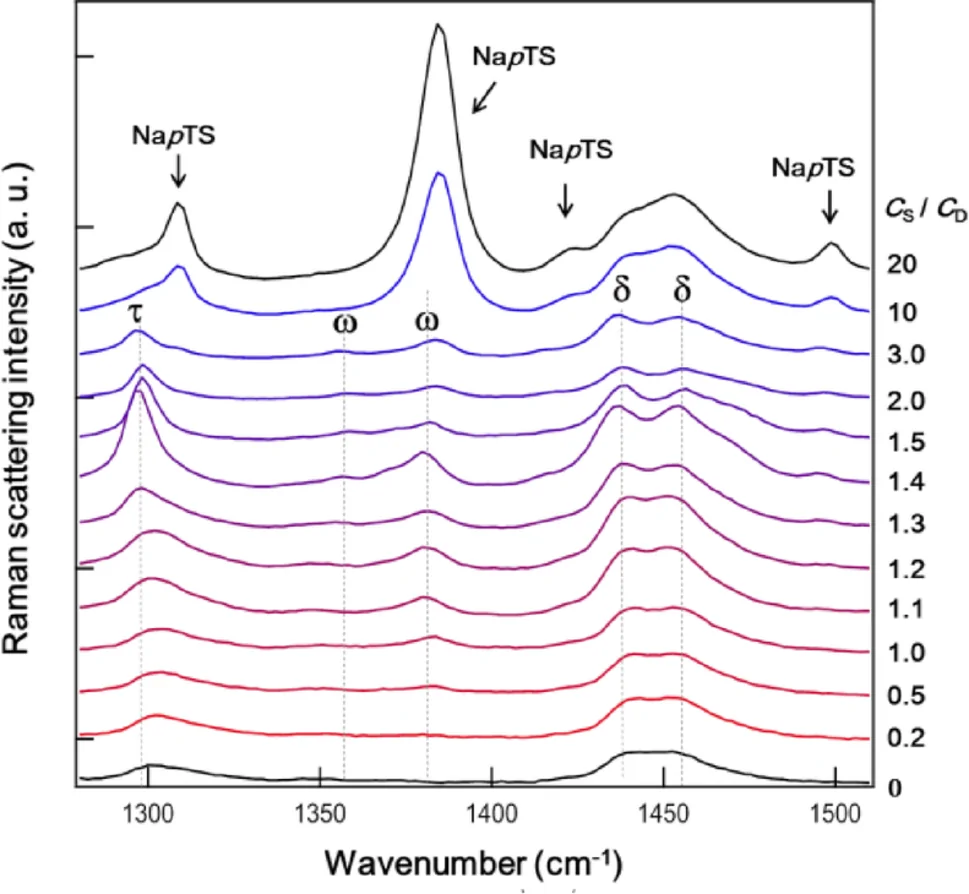
Comparison of the Raman spectra of the CTAB- Na*p*TS system at *C*_S_/*C*_D_ ratios ranging from 0.0 to 20. Dotted lines indicate are included to highlight the wavenumber shifts of the CH_2_ twisting (τ) and scissoring (δ) bands. The two dotted lines at 1360 cm^− 1^ and 1380 cm^− 1^ indicate the expected wavenumbers of the CH_2_ wagging modes (ω). At *C*s/*C*d = 10 and 20, the Raman bands of Na*p*TS at 1310, 1385, 1425, and 1500 cm^− 1^ make significant contributions to the spectra and should therefore be considered in the analysis

With further increasing in salt/surfactant ratio (*C*_S_/*C*_D_), further elongation of the cylindrical micellar region can be expected, corresponding to a transition from short, rigid rod-like micelles to long, flexible worm-like micelles. Such elongation is like to be accompanied by structural undulation, resulting in the increased heterogeneity of the local alkyl-chain packing due to enhanced curvature and chain disorder compared with ideal cylinder regions. Figure 4 presents the evolution of the spectral changes in at higher *C*_S_/*C*_D_ ratios up to 20. For the CTAB (50 mM) - Na*p*TS system, entanglement of the elongated worm-like micelles and the resulting viscoelastic behavior were reported up to *C*_S_/*C*_D_ = 2.0 based on Rheo-SANS measurements [21]. Therefore, the data obtained here for *C*_S_/*C*_D_ = 3.0, 10, and 20 are used to evaluate the upper limit of the persistence continuation of spectral features associated with worm-like micelles, as well as the emergence of dominant Raman features from Na*p*TS in the presence of excess salt. As observed, the spectral features at *C*_S_/*C*_D_ = 3.0 are similar to those at *C*_S_/*C*_D_ = 2.0. However, Raman bands originating from Na*p*TS become dominant at Cs/Cd = 10 and 20, where the possibility of formation additional aggregates cannot be ruled out. Therefore, in the following discussion, we focused on the Raman spectra up to *C*_S_/*C*_D_ = 2.0, where the formation of worm-like micelles has been reported by Rheo-SANS and SLS measurements [21].

**Fig. 5:**
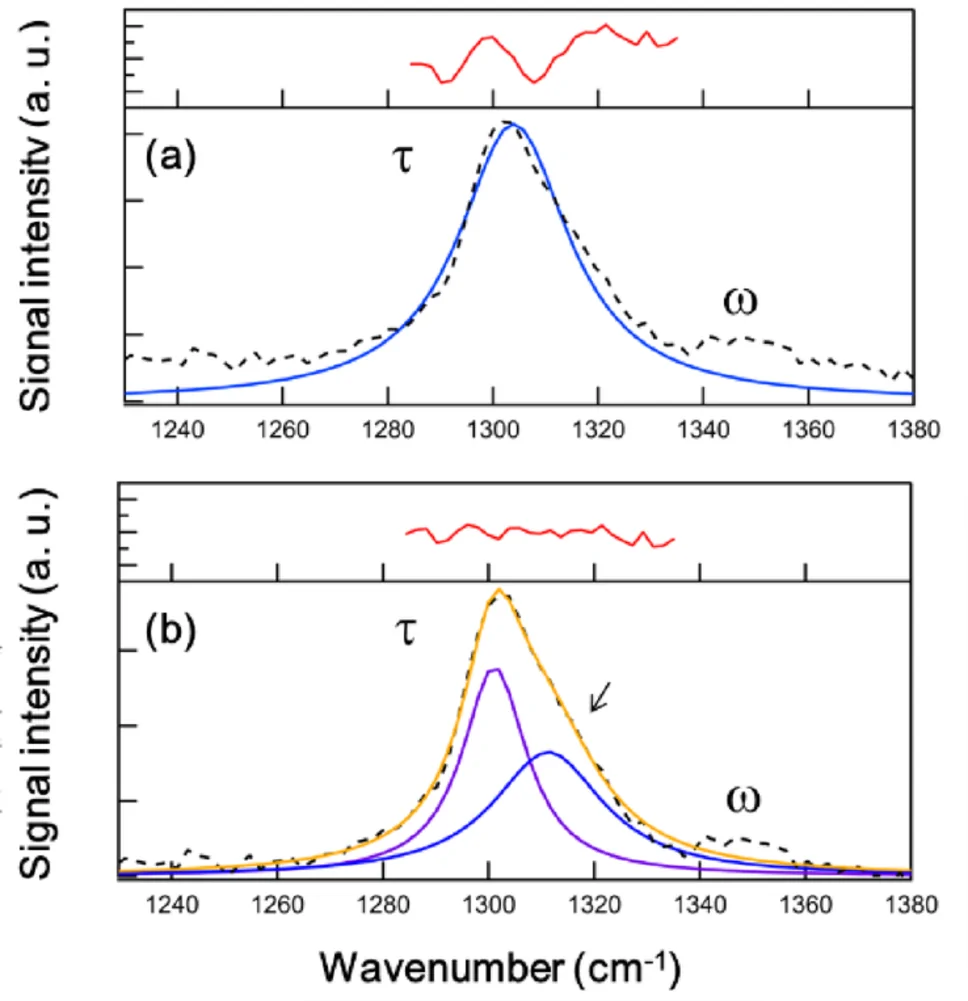
Curve-fitting analysis of the CH_2_ twisting mode (τ) of CTAB (50 mM) using a single Lorentzian function and a sum of two Lorentzian functions. The fitting was performed over the range of 1245–1335 cm^− 1^ to avoid the CH_2_ wagging band (ω) appearing at 1340–1360 cm^− 1^. In each panel, the corresponding residual is shown in the upper section. The dotted lines represent the experimental spectra. The arrow in the lower panel indicates a shoulder in the twisting band. **a** The single Lorentzian function produces systematic residuals on both the lower- and higher-wavenumber sides of the peak. **b** The sum of two Lorentzian functions provides an excellent fit to the experimental spectrum. The fitting parameters obtained from the two-component Lorentzian model for four independent measurements (*N* = 4), with the total integrated area normalized to 1.000, are as follows: (1) Lower-wavenumber component: peak position = 1300.6 ± 0.35 cm^− 1^, FWHM = 14.09 ± 0.74 cm^− 1^, normalized area = 0.467 ± 0.075; (2) Higher-wavenumber component: peak position = 1310.6 ± 1.10 cm^− 1^, FWHM = 23.09 ± 1.12 cm^− 1^, normalized area = 0.533 ± 0.075

To obtain further quantitative indices for detecting morphological transition, we analyzed the CH_2_ twisting and scissoring modes by curve fitting analysis. We first examined the CH_2_ twisting mode of the CTAB solution without Na*p*TS, which represent the local packing environment of alkyl chains in spherical micelles (Fig. 5). The Raman band at around 1300 cm^-1^ was initially fitted with a single Lorentzian function. However, the CH_2_ twisting band exhibited a noticeable asymmetry even at *C*s/*C*d = 0.0. Consequently, fitting with a single Lorentzian function resulted in systematic deviations, as evidenced by the residual spectrum shown in the upper panel of Fig. 5. The spectra were therefore with the sum of two Lorentzian functions because a distinct shoulder was observed on the higher-wavenumber side of the CH_2_ twisting band. This two component model reproduced the experimental spectra with substantially improved accuracy. At first glance, one might expect spherical micelles to provide a relatively uniform packing environment in their hydrophobic core because of their overall structural symmetry. However, the Raman spectra in the 1000 cm^-1^ to 1200 cm^-1^ region exhibit multiple bands arising from the trans and gauche conformers of the alkyl chains in spherical micelles as shown in the upper panel in Fig. 1. This observation indicates that the hydrophobic core contains at least two distinct local conformational environments associated with trans (extended) and to gauche (kinked) alkyl chain segments. These different C–C skeletal conformations are expected to give rise to distinct local packing environments, including variations in intermolecular spacing and in the local steric constraints experienced by the CH_2_ groups during the twisting, wagging, and scissoring vibrations. Consequently, even for spherical micelles, at least two characteristic vibrational frequencies are expected to contribute to the CH_2_ twisting band, providing a physical basis for the successful fitting of the band with two Lorentzian components.

**Fig. 6:**
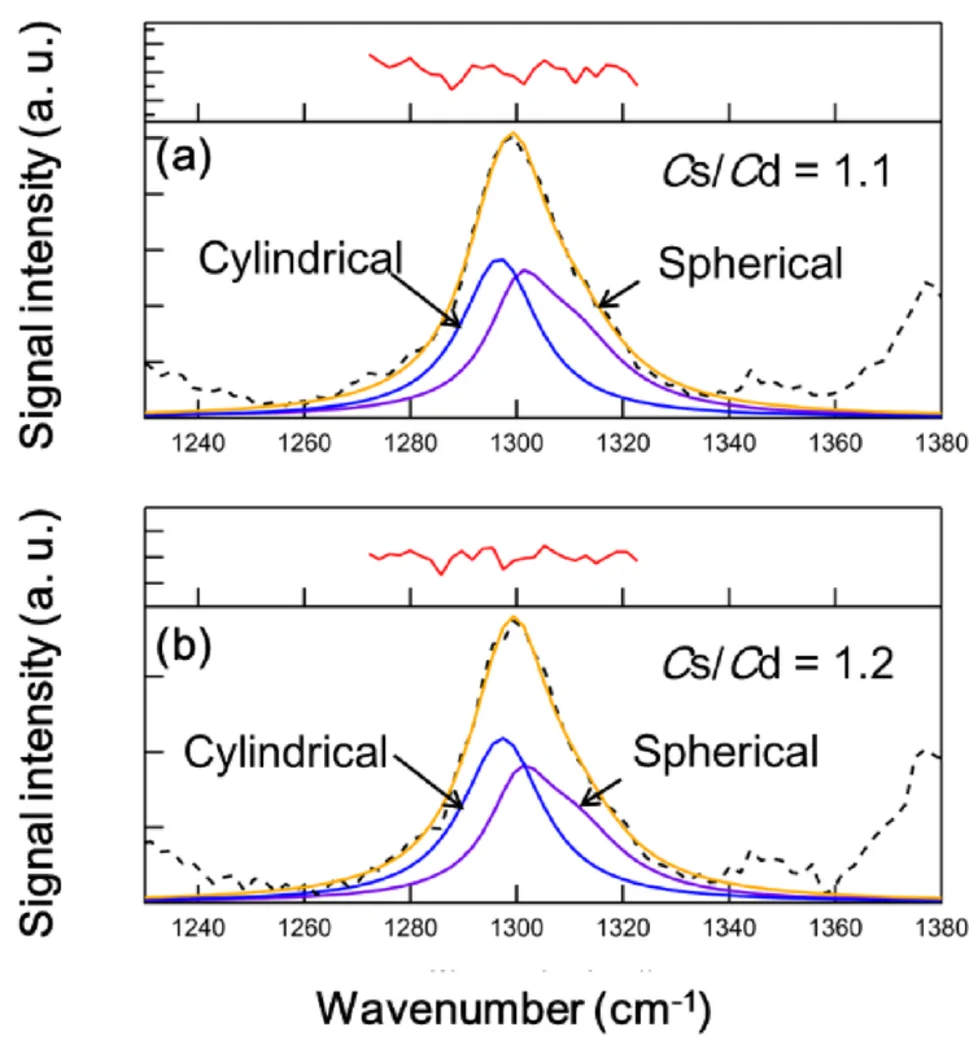
Curve-fitting analyses of the CH_2_ twisting band (τ) of CTAB (50 mM) at *C*s/*C*d = **a** 1.1 and **b** 1.2 using an additional Lorentzian component on the lower-wavenumber side of the model for spherical micelles. The fitting was performed over the wavenumber range of 1280–1330 cm^− 1^. The corresponding residual for each fit is shown in the upper part of the each spectrum. Fitting parameters are summarized in Table 1 in the main text

**Fig. 7:**
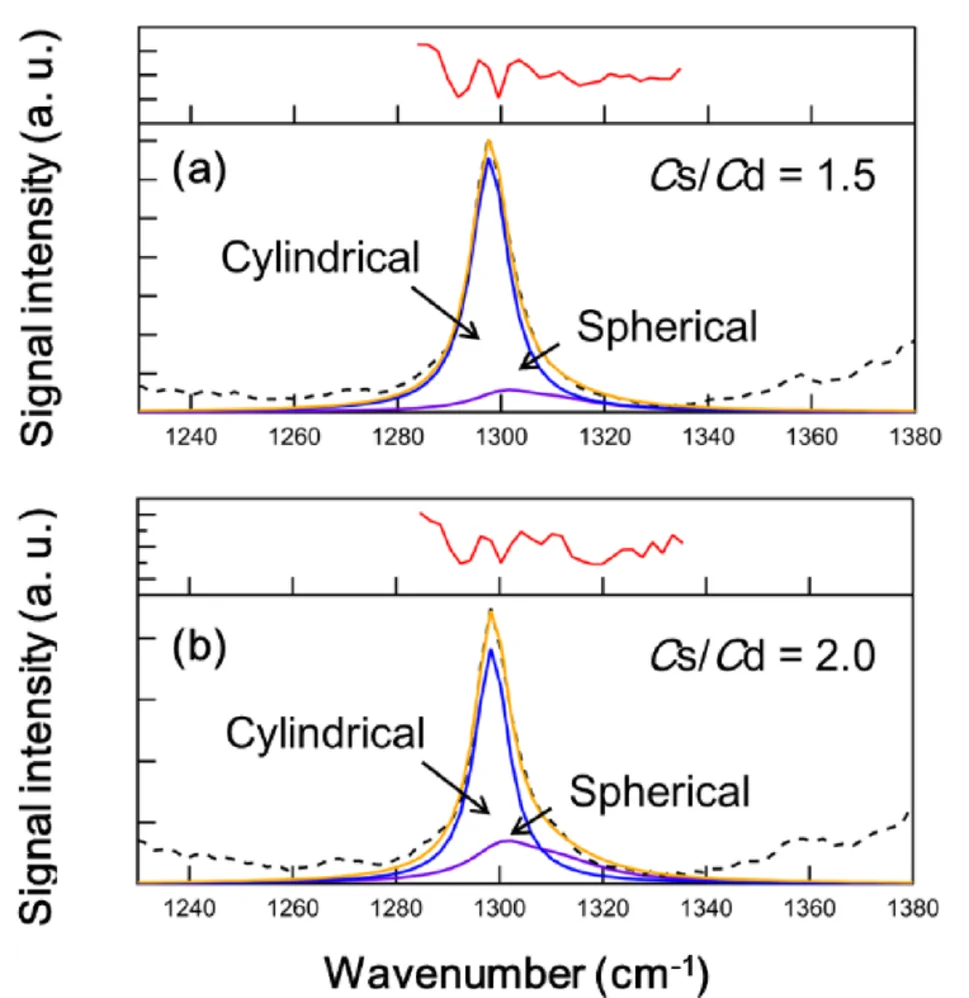
Curve-fitting analyses on the CH_2_ twisting band (τ) of CTAB (50 mM) at *C*s/*C*d = **a** 1.5 and **b** 2.0 using an additional Lorentzian component on the lower-wavenumber side to the model for spherical micelles. The fitting was performed over the wavenumber range of 1280–1330 cm^− 1^. The corresponding residual for each fit is shown in the upper part of each spectrum. Fitting parameters are summarized in Table 1 in the main text

To evaluate the emergence and elongation of the cylindrical domains in the rod- and worm-like micelles, we analyzed the Raman spectra by introducing an additional spectral component to the model used for the spherical micelles. The spectra of the spherical micelles were represented by two Lorentzian components (higher- and lower-components), whereas an additional component was introduced for samples with *C*s/*C*d > 1.0, as summarized in Table 1. This approach was adopted because Takeda et al. reported a remarkable change in the viscoelastic property at Cs/Cd > 1.0 [21], and similarly, the CH_2_ twisting band measured in the present study also exhibited marked spectral changes above this ratio. During the spectral fitting, the relative area of the two Lorentzian components assigned to the spherical micelles was fixed, and only a single additional component was introduced to account for the emergence of a different local environment, which was assigned to the cylindrical domain. It should be noted that even in the rod- and worm-like micelles are expected to retain hemispherical end caps that shield the hydrophobic core of the cylindrical domain from the surrounding aqueous phase. Therefore, a spherical contribution should remain even after the micelles transform into rod- or worm-like structures. As shown in Table 1, the spectra at *C*s/*C*d = 1.3 and 1.4 exhibit transitional characteristics, where the full width at half maximum (FWHM) of the additional component decreased markedly from approximately 20 cm^-1^ to 10 cm^-1^. Based on the distinct evolution of the spectral components with increasing *C*s/*C*d, we propose that the appearance of the additional component on the slightly lower-wavenumber side of the CH_2_ twisting band of the spherical micelles can serve as spectroscopic indicator for the emergence of cylindrical domains, i.e. the formation of rod-like micelles (under the present experimental conditions, observed at *C*s/*C*d = 1.1 and 1.2; Fig. 6). Furthermore, pronounced increase in the relative intensity of this additional component, together with its significant narrowing, can be used as an indicator of the growth of the cylindrical domains from rod-like to worm-like micelles (under the present experimental conditions, observed at *C*s/*C*d = 1.5 and 2.0, Fig. 7). The substantial increase in the peak area of the additional component relative to that of the spherical components strongly supports the elongation of the cylindrical domains and the formation of worm-like micelles. It should be also noted that the relative area of the spherical domains, reference to the spectrum at *C*s/*C*d = 0, exhibit a slight increase again at *C*s/*C*d = 1.5 to 2.0. This trend may reflect an increase in the number of curved or winding regions within the elongated cylindrical structures of the worm-like micelles.

**Table 1:** Peak frequency, full width at half maximum (FWHM), and relative area of an additional Lorentzian component on the lower-wavenumber side of the model for spherical micelles

Cs / Cd	Peak Frequency / cm^− 1^		FWHM / cm^− 1^		Relative Area*		Spherical (lower comp.)		Spherical (higher comp.)	
	Ave.*	Std.*	Ave.	Std.	Ave.	Std.	Ave.	Std.	Ave.	Std.
1.1	1296.29	± 0.66	17.01	± 1.70	0.442	± 0.057	0.257	± 0.026	0.300	± 0.030
1.2	1298.14	± 0.55	19.66	± 0.80	0.625	± 0.077	0.173	± 0.036	0.202	± 0.041
1.3	1300.91	± 0.81	20.75	± 0.49	0.605	± 0.054	0.182	± 0.025	0.212	± 0.029
1.4	1294.84	± 0.63	11.95	± 3.14	0.815	± 0.161	0.085	± 0.075	0.099	± 0.087
1.5	1297.87	± 0.06	8.95	± 0.28	0.792	± 0.057	0.096	± 0.026	0.112	± 0.031
2.0	1298.58	± 0.05	8.57	± 0.47	0.697	± 0.033	0.140	± 0.015	0.163	± 0.018

*Ave.: Average for *N* = 4 data. Std.: Standard deviation of *N* = 4 data. Relative Area: Normalized with the total area of the CH_2_ twisting band (an additional component and the lower- and higher- wavenumber components for spherical micelles = 1.000). Peak frequencies, band widths, and relative peak area of the lower- and higher-wavenumber components for spherical micelles are fixed for all fittings ranging from *C*s/*C*d = 1.1 to 2.0

**Fig. 8:**
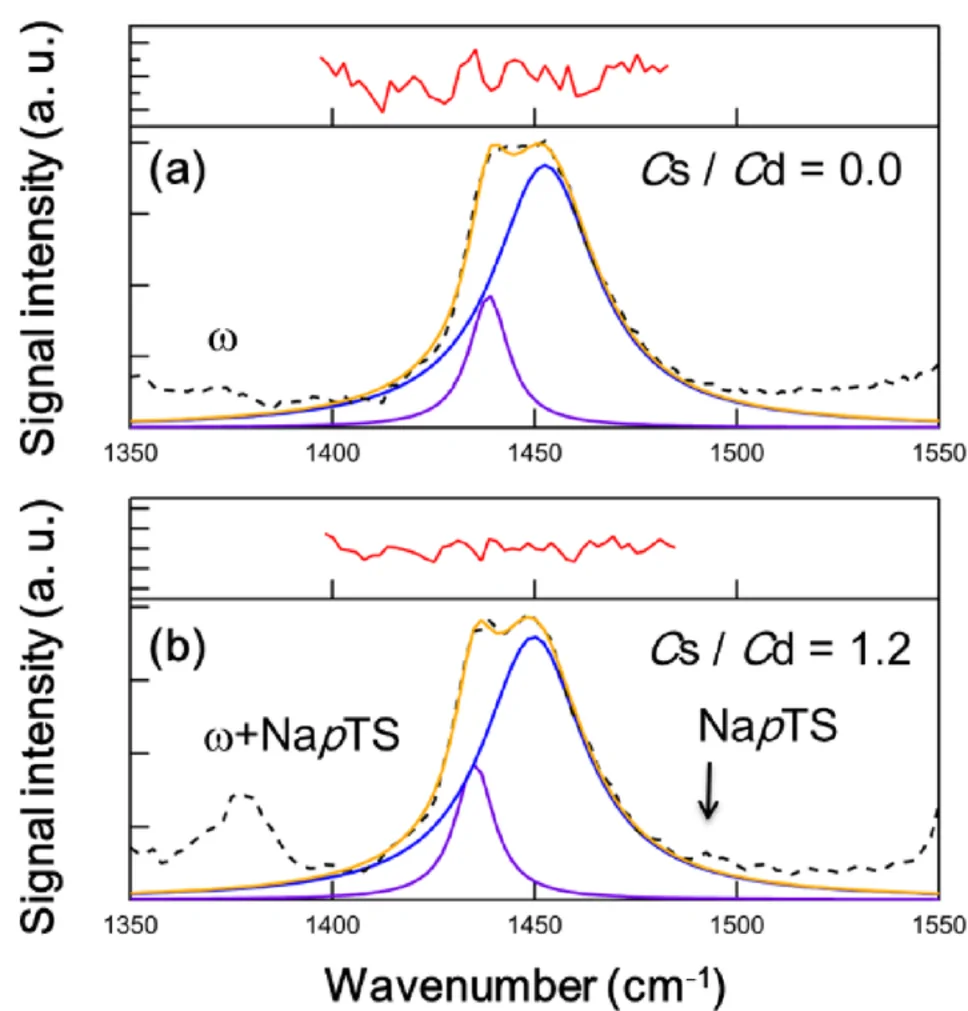
Curve-fitting analyses of the CH_2_ scissoring mode (δ) of CTAB (50 mM) at *C*s/*C*d = **a** 0 (spherical micelles) and **b** 1.2 (rod-like micelles) using a sum of two Lorentzian functions. The fitting parameters are summarized in Tables 2, 3 and 4 in the main text

**Fig. 9:**
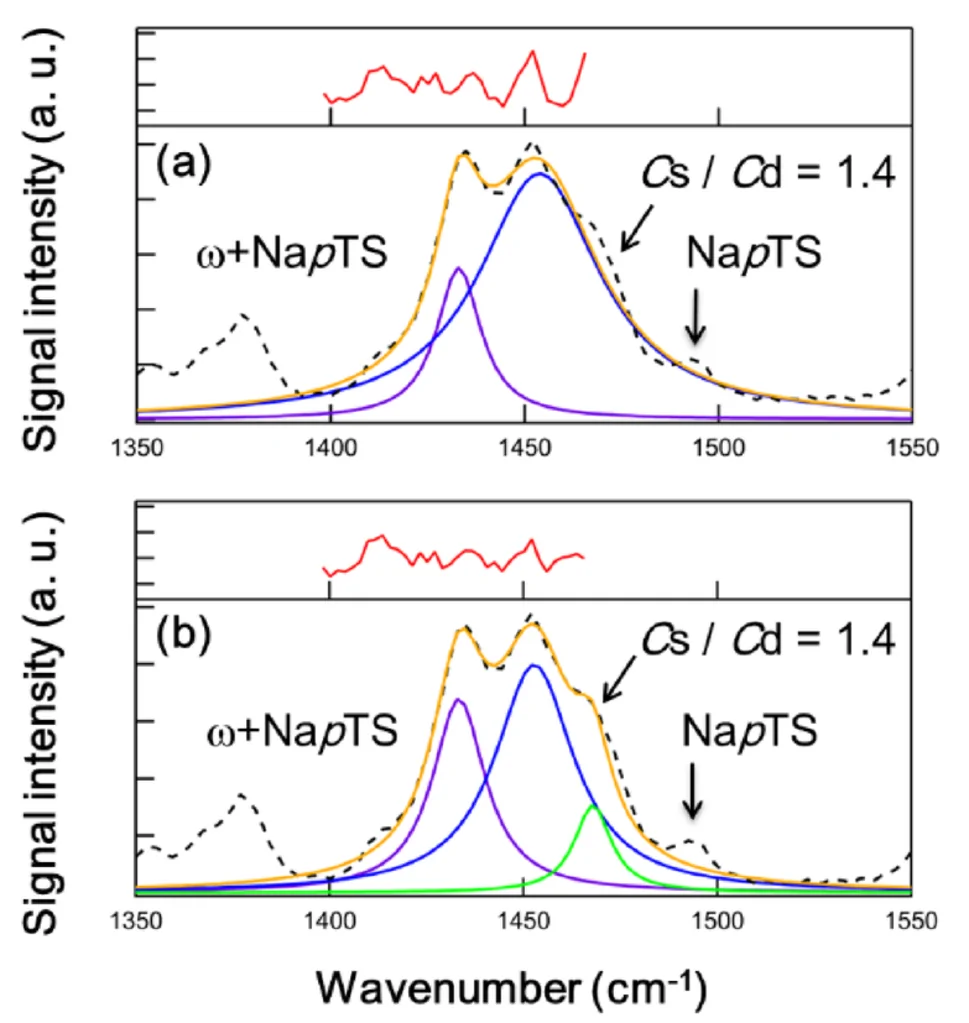
Curve-fitting analyses of the CH_2_ scissoring mode (δ) of CTAB (50 mM) at *C*s/*C*d = 1.4 (the ratio of the transition from rod-like to worm-like micelles) using **a** the sum of tow Lorentzian functions and **b** the sum of three Lorentzian functions. Allows in the figures indicate the appearance of shoulder features on the higher-wavenumber side of the CH_2_ scissoring band. The fitting parameters for the Lorentzian functions are summarized in the Tables 2, 3 and 4 in the main text. The Raman peaks at approximately 1380 and 1495 cm^− 1^, which originated from Na*p*TS, increase in intensity with increasing Na*p*TS concentration

To support the proposed assignments described above and to obtain further spectral indicators of the morphological transition of micelles from spherical to rod-like and worm-like structures, we investigated the changes in the Raman spectra of the CH_2_ scissoring modes as the *C*s/*C*d ratio increased. As mentioned above, the CH_2_ twisting vibration occurs along the C-C skeletal axis, whereas the CH_2_ scissoring vibration occurs perpendicular to the C-C skeletal axis. Therefore, the CH_2_ scissoring mode is also expected to sensitively reflect changes in the lateral packings of neighboring alkyl chains induced by bending of the cylindrical region of worm-like micelles, compared with that of the shorter and rigid rod-like micelles. First, the Raman spectra for *C*s/*C*d = 0 and *C*s/*C*d = 1.2 were compared (Fig. 8). The former corresponds to spherical micelles, whereas the latter corresponds to rod-like micelles. As shown in Figs. 3 and 4, the CH_2_ scissoring band consists of at least two components. As expected, fitting with a double-Lorentzian function successfully reproduced the experimental spectra for both *C*s/*C*d = 0 and 1.2. The fitting parameters are summarized in Table 2 (peak wavenumbers), Table 3 (full width at half maximum, FWHM), and Table 4 (relative areas of individual components). In contrast to the changes observed in the CH_2_ twisting band between *C*s/*C*d = 0 and 1.2, only minor changes were found in the spectral features of the CH_2_ scissoring band. As shown in Figs. 4, Raman peaks originating from Na*p*TS appear at approximately 1310, 1385, 1425, and 1500 cm^− 1^ for *C*s/*C*d > 10. In particular, the Raman peak at 1500 cm^− 1^ becomes noticeable at *C*s/*C*d ≥ 1.2. Therefore, the overlap of these Na*p*TS peaks with those arising from CTAB molecules should be carefully taken into account in the spectral analysis at higher *C*s/*C*d ratios. At *C*s/*C*d = 1.4, a shoulder structure appears on the higher-wavenumber side of the CH_2_ scissoring peak (indicated by the arrow in Fig. 9). As mentioned above, *C*s/*C*d = 1.4 has been proposed as the transition ratio from rod-like to worm-like micelles based on the analysis on the CH_2_ twisting peak. Therefore, the appearance of this shoulder component is expected to serve as a useful indicator for detecting the emergence of the worm-like micelles. The double- and triple-Lorentzian fitting results for the Raman spectrum at *C*s/*C*d = 1.4 are compared in Fig. 9; Tables 2, 3 and 4. The newly introduced Component 3, located at the highest wavenumber (approximately 1470 cm^− 1^), successfully accounts for the shoulder feature together with Component 1 (approximately 1435 cm^− 1^) and Component 2 (approximately 1455 cm^− 1^).

**Table 2:** Peak frequencies of the fitting components for CH_2_ scissoring band with the sum of two or three Lorentzian functions

Cs / Cd	Component 1 / cm^− 1^		Component 2 / cm^− 1^		Component 3 / cm^− 1^	
	Ave.	Std.	Ave.	Std.	Ave.	Std.
0	1438.8	± 0.11	1452.5	± 0.09	-	-
1.1	1435.3	± 0.06	1450.0	± 0.15	-	-
1.2	1435.3	± 0.10	1449.9	± 0.12	-	-
1.3	1437.4	± 0.23	1452.4	± 0.08	-	-
1.4	1433.2	± 1.18	1452.7	± 1.25	-	-
1.4(※)	1433.6	± 1.22	1451.4	± 0.96	1464.0	± 4.36
1.5	1436.9	± 0.39	1455.1	± 0.31	1468.8	± 0.65
2.0	1437.8	± 0.46	1456.1	± 0.34	1469.8	± 1.23

**Fig. 10:**
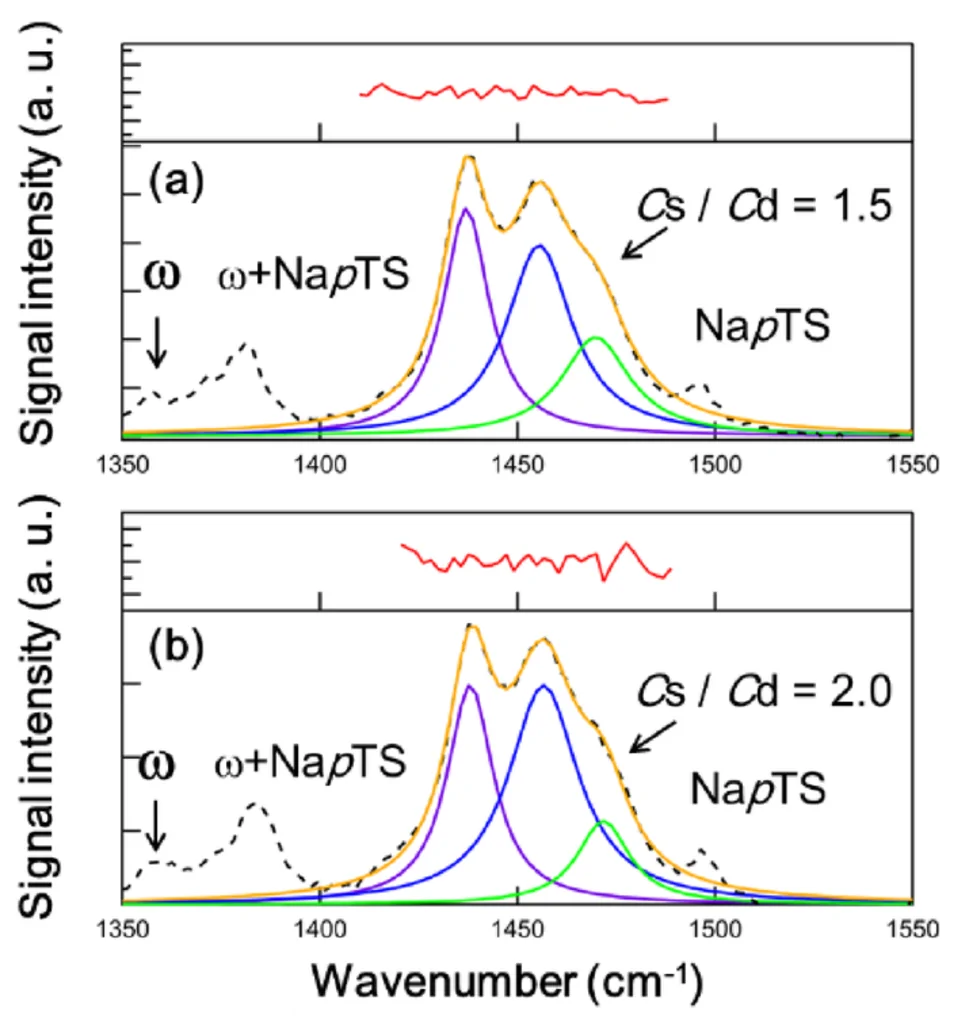
Curve-fitting analyses of the CH_2_ scissoring mode (δ) of CTAB (50 mM) at *C*s/*C*d = **a** 1.5 and **b** 2.0 with the sum of three Lorentzian functions, where the predominance of worm-like micelles was suggested by the analysis of CH_2_ twisting mode. The arrows in the figures indicate the shoulder features on the higher-wavenumber side of the CH_2_ scissoring band, which are also observed at rod-like-to-worm-like transition ratio (*C*s/*C*d = 1.4). The fitting parameters for the Lorentzian functions are summarized in Tables 2, 3 and 4 in the main text. The shoulder peak assigned to the CH_2_ wagging mode appears at approximately 1360 cm^− 1^

Ave.: Average of *N* = 4 data at each *C*s/*C*d ratio. Std.: Standard deviation of *N* = 4 data at each *C*s/*C*d ratio. (※) At *C*s/*C*d ratio = 1.4, where error becomes non-negligible, the analyses with both the sum of two and three components are performed. Corresponding curve fittings are shown in Figs. 8 and 9, and 10

**Table 3:** Band widths (FWHM) of the fitting components for CH_2_ scissoring band with the sum of two or three Lorentzian functions

Cs / Cd	Component 1 / cm^− 1^		Component 2 / cm^− 1^		Component 3 / cm^− 1^	
	Ave.	Std.	Ave.	Std.	Ave.	Std.
0	11.20	± 0.47	31.35	± 0.39	-	-
1.1	12.48	± 0.74	30.40	± 0.80	-	-
1.2	11.64	± 0.17	31.40	± 0.45	-	-
1.3	11.54	± 0.16	31.82	± 1.06	-	-
1.4	13.42	± 0.28	32.59	± 2.29	-	-
1.4(※)	15.11	± 1.62	22.59	± 2.56	22.56	± 7.49
1.5	13.99	± 0.14	19.21	± 1.33	20.60	± 4.86
2.0	14.94	± 0.83	19.51	± 1.17	19.07	± 2.31

Ave.: Average of *N* = 4 data at each *C*s/*C*d ratio. Std.: Standard deviation of *N* = 4 data at each *C*s/*C*d ratio. (※) At *C*s/*C*d ratio = 1.4, where error becomes non-negligible, the analyses with both the sum of two and three components are performed. Corresponding curve fittings are shown in Figs. 8 and 9, and 10

**Table 4:** Relative peak areas of the fitting components for CH_2_ scissoring band with the sum of two or three Lorentzian functions

Cs / Cd	Component 1		Component 2		Component 3	
	Ave.	Std.	Ave.	Std.	Ave.	Std.
0	0.145	± 0.011	0.855	± 0.011	-	-
1.1	0.185	± 0.024	0.815	± 0.024	-	-
1.2	0.161	± 0.004	0.838	± 0.004	-	-
1.3	0.165	± 0.009	0.835	± 0.009	-	-
1.4(※)	0.227	± 0.030	0.773	± 0.030	-	-
1.4	0.326	± 0.034	0.465	± 0.009	0.209	± 0.103
1.5	0.359	± 0.010	0.416	± 0.097	0.224	± 0.099
2.0	0.379	± 0.023	0.432	± 0.048	0.189	± 0.026

Ave.: Average of *N* = 4 data at each *C*s/*C*d ratio. The total integrated area is normalized to 1.000. Std.: Standard deviation of *N* = 4 data at each *C*s/*C*d ratio. (※) At *C*s/*C*d ratio = 1.4, where error becomes non-negligible, the analyses with both the sum of two and three components are performed. Corresponding curve fittings are shown in Figs. 8 and 9, and 10

To confirm that the spectral feature appearing at approximately 1470 cm^− 1^ indeed represents the emergence of the worm-like micelles, we further examined the Raman spectra at *C*s/*C*d = 1.5 and 2.0, where the formation of the worm-like micelles has previously been proposed base on SANS/SLS measurements [21] and was also suggested by the changes in the CH_2_ twisting band observed in the present study. As expected, the Raman spectra at *C*s/*C*d = 1.5 and 2.0 both exhibit a significant contribution from Component 3, which accounts for approximately 20% of the total peak area of the CH_2_ scissoring band (Fig. 10; Table 4). Interestingly, the relative peak area of Component 1, located at the lowest wavenumber (approximately 1435 cm^− 1^), also increases from approximately 20% to 35% at *C*s/*C*d = 1.5 and 2.0. In the worm-like morphology, the bent regions of the cylindrical micelles simultaneously create expanded intermolecular spacing on the outer side of the bend and compressed spacing on the inner side, thereby altering the lateral packings of the alkyl chains. Because weaker attractive interactions lead to higher wavenumber vibration, the simultaneous increase in the highest-wavenumber vibration Component 3, corresponding to the outer side of the bent cylinder, and the lowest wavenumber Component 1, corresponding to the inner side, strongly supports the formation of the long, winding worm-like micelles. It is also noteworthy that, at *C*s/*C*d = 1.5 and 2.0, a shoulder band appears of the CH_2_ wagging band at approximately 1360 cm^− 1^ on the lower wave-number side of the main CH_2_ wagging band at approximately 1380 cm^− 1^. Unfortunately, the main band of the CH_2_ wagging at approximately 1380 cm^− 1^ overlaps with a band originated from Na*p*TS in the present system, making it difficult to accurately evaluate the contribution of the shoulder band at 1360 cm^− 1^. Nevertheless, this CH_2_ wagging band may also serve as a useful spectral marker for detecting the emergence of worm-like micelles in other surfactant-salt system. Further investigation of this possibility will be the subject of future work.

## Conclusion

We examined the applicability of Raman scattering spectroscopy for detecting the morphological transitions of micelles from spherical to short, rigid rod-like and long, flexible worm-like structures using the representing CTAB-Na*p*TS system, whose morphologies have been extensively characterized by SANS and SLS measurements. The result demonstrates that the changes in the CH_2_ twisting and scissoring modes provide useful spectral indicators of the morphological transition from spherical to rod-like and worm-like micelles. In particular, the appearance of an additional component in the CH_2_ twisting band at around 1300 cm^− 1^, compared with that of spherical micelles, is proposed as a useful indicator of the emergence of the rod-like morphology. Furthermore, the marked narrowing of the bandwidth of this additional component, from approximately 20 cm^− 1^ to 10 cm^− 1^ (FWHM), provides a useful signature for detecting the morphological transition from rod-like to worm-like micelles. During the rod-like-to-worm-like transition, which occurs at *C*s/*C*d = 1.4, a new component also appears in the CH_2_ scissoring band as a shoulder at around 1470 cm^− 1^. The simultaneous increases in the peak areas of the highest- (1470 cm^− 1^) and lowest-wavenumber (1435 cm^− 1^) components observed at *C*s/*C*d = 1.5 and 2.0 suggest that these components reflect the coexistence of locally widened and narrowed lateral packing of alkyl chains induced by the bending of cylindrical regions of the worm-like micelles. The appearance of new components in the CH_2_ twisting and scissoring bands, together with the accompanying changes in peak position and bandwidth, is robust against variations in Raman signal intensities caused by changes in additive concentration. These Raman spectroscopic signatures should therefore provide useful spectral indicators for process monitoring under atmospheric and open-system conditions, where the additive concentration and solvent content can vary continuously. Such conditions are commonly encountered during the production of foods and pharmaceuticals in on-site process control of inkjet printing and enhanced oil recovery.

## Data Availability

The datasets analyzed during the current study are available from the corresponding author on reasonable request.
